# Supplementary material for: A Critical View of the Application of the APEX Software (Aqueous Photochemistry of Environmentally-Occurring Xenobiotics) to Predict Photoreaction Kinetics in Surface Freshwaters
Source: Molecules. 2019 Dec 18;25(1):9. doi: 10.3390/molecules25010009 (PMC7017383; doi:10.3390/molecules25010009)
Supplement: Supplementary file 1 [file molecules-25-00009-s001.zip › 3.2.4_gcc-4.4.0.zip!/3.2.4_gcc-4.4.0/3.2.4_gcc-4.4.0/doc/octave/html/interpreter/Other-Output-Conversions.html]

Other Output Conversions - Untitled


Next: Formatted Input,
Previous: Floating-Point Conversions,
Up: C-Style I/O Functions


---

#### 14.2.10 Other Output Conversions

This section describes miscellaneous conversions for `printf`.

The %c conversion prints a single character. The -
flag can be used to specify left-justification in the field, but no
other flags are defined, and no precision or type modifier can be given.
For example:

```
     printf ("%c%c%c%c%c", "h", "e", "l", "l", "o");
```

prints hello.

The %s conversion prints a string. The corresponding argument
must be a string. A precision can be specified to indicate the maximum
number of characters to write; otherwise characters in the string up to
but not including the terminating null character are written to the
output stream. The - flag can be used to specify
left-justification in the field, but no other flags or type modifiers
are defined for this conversion. For example:

```
     printf ("%3s%-6s", "no", "where");
```

prints  nowhere  (note the leading and trailing spaces).
